# Impact of educational level and travel burden on breast cancer stage at diagnosis in the state of Sao Paulo, Brazil

**DOI:** 10.1038/s41598-022-12487-9

**Published:** 2022-05-19

**Authors:** Raissa Janine de Almeida, Carolina Terra de Moraes Luizaga, José Eluf-Neto, Hélio Rubens de Carvalho Nunes, Eduardo Carvalho Pessoa, Cristiane Murta-Nascimento

**Affiliations:** 1 Faculty of Medicine, Sao Paulo State University (UNESP), Unesp. Av. Prof. Montenegro, s/n, Distrito de Rubião Junior, Botucatu, SP 18618-687 Brazil; 2 Department of Information and Epidemiology, Fundação Oncocentro de São Paulo (FOSP), Sao Paulo, SP Brazil; 3 Department of Preventive Medicine, Faculdade de Medicina FMUSP, Universidade de São Paulo, Sao Paulo, SP Brazil

**Keywords:** Breast cancer, Cancer epidemiology

## Abstract

We describe the characteristics of cases of breast cancer among women assisted at hospitals affiliated to the public health system in the state of São Paulo (Brazil), analysing the effects of level of education and travel burden to point of treatment. We conducted a retrospective analysis of invasive breast cancer among women diagnosed between 2000 and 2015. Data were extracted from the hospital-based cancer registries of Fundação Oncocentro de São Paulo—FOSP. The outcome was clinical stage at diagnosis (stage III–IV versus I–II). The explanatory variables were educational level and travel burden. Odds ratios (OR) and 95% confidence intervals (95% CI) were estimated. Multiple imputations were used for missing educational level (31%). The study included 81,669 women with invasive breast cancer diagnosed between 2000 and 2015. The mean age of patients at diagnosis was 56.8 years (standard deviation 13.6 years). 38% of patients were at an advanced stage at diagnosis (stage III–IV). Women with lower levels of education and those who received cancer care in municipalities other than where they lived were more likely to be diagnosed at an advanced stage. In conclusion, promotion of breast cancer awareness and improving pathways to expedite breast cancer diagnosis and treatment could help identify breast tumors at earlier stages.

## Introduction

Breast cancer (BC) is a major public health concern, and it is the most common malignancy among women worldwide. Over 2.2 million new cases of BC were estimated for 2020, accounting for 24.5% of all cancers diagnosed in women. BC is also the leading cause of death due to cancer among women globally (684,996 deaths estimated in 2020)^1^.

In Brazil, BC is the most common malignancy among women after nonmelanoma skin cancer. The Brazilian National Cancer Institute (INCA) estimated that around 66,200 new BC cases among women are diagnosed in Brazil each year (2020–2022), resulting in an age-standardized incidence rate of 43.74/100,000 women^2^. Regarding mortality, there were over 18,000 deaths due to BC among women in 2019 (age-standardized death rate of 14.23/100,000 women) (available at http://mortalidade.inca.gov.br/Mortalidade). Moreover, 18,280 new cases of BC are estimated to occur in the state of São Paulo each year (2020–2022) (age-standardized incidence rate of 55.4/100,000 women)^2^.

When diagnosed early, BC is a potentially curable disease. Poor BC survival rates observed in low- and middle-income countries are mainly related to the high proportion of advanced stage BC at diagnosis in these countries. A systematic review published in 2016 reported that, in sub-Saharan African countries, the median prevalence of advanced stage at presentation was 74.7%, ranging from 30.3 to 100%^3^. More recently, in India, approximately 67% of BC patients were diagnosed with locoregional or metastatic disease^4^. By comparison, approximately 13% of female BC cases in the US were diagnosed at stage III or IV between 2004 and 2016^5^, and in a study performed in two Swedish health regions between 1989 and 2013 (n = 42,220), the prevalence of stage III/IV at diagnosis was 17%^6^. In Brazil, 40.2% of BC cases diagnosed between 2001 and 2014 and included in the nationwide hospital-based cancer registry (HBCR) network were at stage III or IV at diagnosis^7^.

The Breast Health Global Initiative has pointed out the need to promote early diagnosis of symptomatic disease in low- and middle-income countries as it will improve outcomes for many women with BC^8^. Identifying addressable circumstances that lead to advanced stages at diagnosis should be a priority in those countries. Recently, Dos-Santos-Silva and cols assessed factors associated with late-stage BC at diagnosis in Brazil^7^. They identified a higher chance of advanced BC stage among black and brown women, or with a lower level of education. Information regarding the state of São Paulo was not included in some of their analyses because HBCRs in São Paulo do not always collect the same information as in other states (e.g., ethnicity).

Other than that, studies performed in other countries obtained inconsistent results when assessing geographic effects on access to health services for appropriate diagnosis and treatment^9,10^. In Brazil, as far as we know, no previous study ever sought to analyse the effect of stage of BC at diagnosis among patients receiving cancer care outside their municipality of residence.

Thus, the present study aimed to describe the main characteristics of the female BC population assisted in hospitals affiliated to the public health system in the state of São Paulo (Brazil), and to assess the impact of level of education and travel burden on BC stage at diagnosis in this population.

## Methods

### Study population and data source

The HBCR network coordinated by the Fundação Oncocentro of São Paulo (FOSP) was used to identify BC cases included in this analysis. The FOSP-HBCR network was established in 2000 to include all hospitals with oncology accreditation affiliated to the Brazilian National Health System (SUS) in the state of São Paulo. The state of São Paulo is located in the Southeast region of Brazil and had an estimated population of 46,289,333 inhabitants in 2020. It has a 248,220 km^2^ area that comprises 645 municipalities (available at https://www.ibge.gov.br/en/cities-and-states/sp.html).

The current number of hospitals included in the FOSP-HBCR network, which has changed over time, is 77, of which 72 are affiliated to the SUS, and five are private institutions that agreed to report cancer cases to the network. The FOSP-HBCR includes all primary neoplasms whose behaviour codes are 1, 2, or 3 according to the International Classification of Diseases for Oncology, 3rd Edition (ICD-O-3). The FOSP-HBCR offers high-quality data indices: % of cases checked microscopically (98.3%), % of cases with missing stage information (4.2%), % of morphology coded as 'Neoplasm, malignant' (0.9%), % of topography coded as 'Malignant neoplasm without specification of the site’ (1.4%), and % of topography coded as 'Malignant neoplasm of other and ill-defined sites’ (0.4%) (available at http://www.fosp.saude.sp.gov.br:443/epidemiologia/docs/Dados_de_Cancer.pdf). The FOSP-HBCR network collects information using software developed by the FOSP. Quality and duplicate checks are performed periodically by the FOSP staff, and when required, lists of errors are sent back to the HBCR for clarifications and corrections.

**Figure 1:**
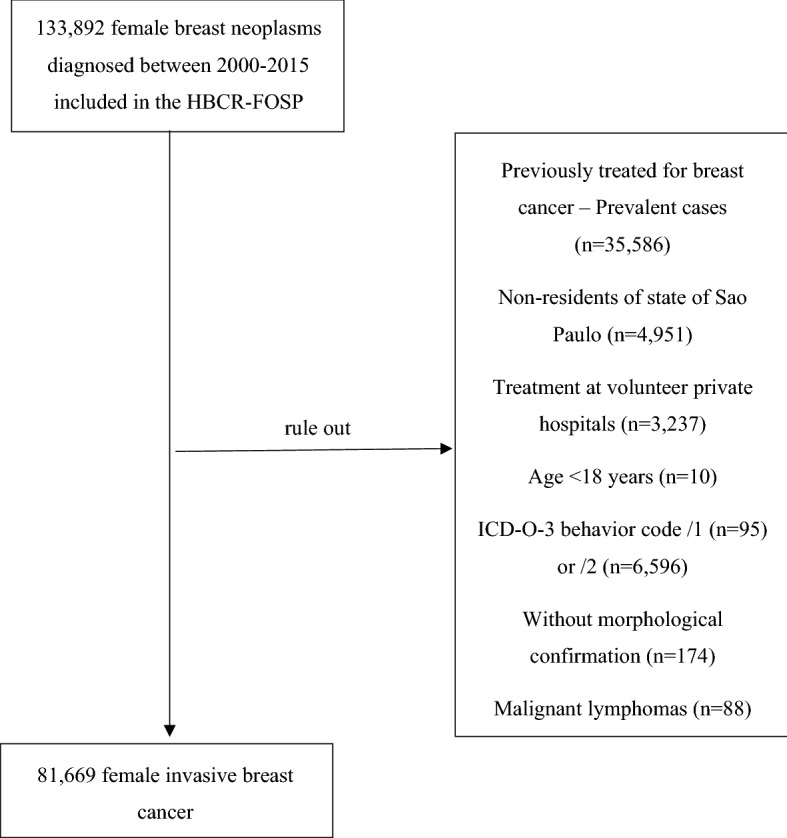
Flow diagram of inclusion criteria and exclusion criteria.

This study included women with invasive BC (International Classification of Diseases, tenth revision [ICD-10]: C50) aged 18 years and above, living in the state of São Paulo, diagnosed between 2000 and 2015, regardless of the TNM classification of malignant tumours (clinical stage I–IV), and starting treatment at one of the hospitals included in the FOSP-HBCR network. Patients without morphological confirmation, those with malignant lymphomas, and those who received oncologic care at the five private hospitals that report cancer cases to FOSP were not included in the analysis. Figure 1 shows inclusion and exclusion criteria.

We extracted all individual characteristics (age at diagnosis, year of diagnosis, level of education, municipality of residence, cancer care site) as well as tumor characteristics (clinical stage at diagnosis and histology type) from the FOSP-HBCR. Outcome was dichotomized as advanced stage (stage III–IV) versus early-stage BC (stage I–II). Covariates studied were level of education and travel burden. Patients were classified into five categories according to level of education: illiterate, less than middle school, middle school, high school, and university. The travel burden variable was dichotomized as follows: (i) cancer care at patient’s municipality of residence and (ii) cancer care at another municipality. In this study, cancer care site refers to the hospital where BC patients received their first treatment, which on many occasions was also the place of diagnosis. Age at diagnosis, year of diagnosis, and histology type were considered confounding factors. Six age groups were considered: 50–59 years and 60–69 years, as screening-exposed age groups, and < 40 years, 40–49 years, 70–79 years, and > 80 years, as screening non-exposed groups.

### Statistical analysis

First, a descriptive analysis was performed including all subjects. The distribution of stage at diagnosis was examined by patient and tumor characteristics. Subsequently, the association between stage at diagnosis (stage III–IV versus stage I–II) and covariates was assessed. Multiple logistic regression was used to estimate odds ratios (OR) and 95% confidence intervals (95% CI). Given one covariate (level of education) presented incomplete information to a large extent, the missing data imputation technique was used to assess the robustness of our results. Missing data were considered to be missing at random (MAR). To impute values of level of education, we used a chained equation procedure to generate 50 sets of plausible values for level of education, based on an imputation model. The imputation model was built using clinical stage, age at diagnosis, year of diagnosis, histology type, and need to travel for cancer care. Statistical significance was set at p < 0.05. Statistical analyses were performed with Stata version 16.1.

### Ethics

This study is in compliance with the ethical precepts of Resolution # 466/2012 on research involving human beings, of the Brazilian National Health Council, and was approved with waiver of the informed consent by the Human Research Ethics Committee of the Medical School of São Paulo State University—Unesp (CAAE: 88216418.8.0000.5411).

## Results

The analysis included 81,669 women with invasive BC diagnosed between the years 2000 and 2015. The mean age at BC diagnosis was 56.8 years (standard deviation 13.6 years). 21.9% of women were diagnosed in stage I (n = 17,878), 40.0% in stage II (n = 32,642), 28.4% in stage III (n = 23,211) and 9.7% in stage IV (n = 7938).

**Figure 2:**
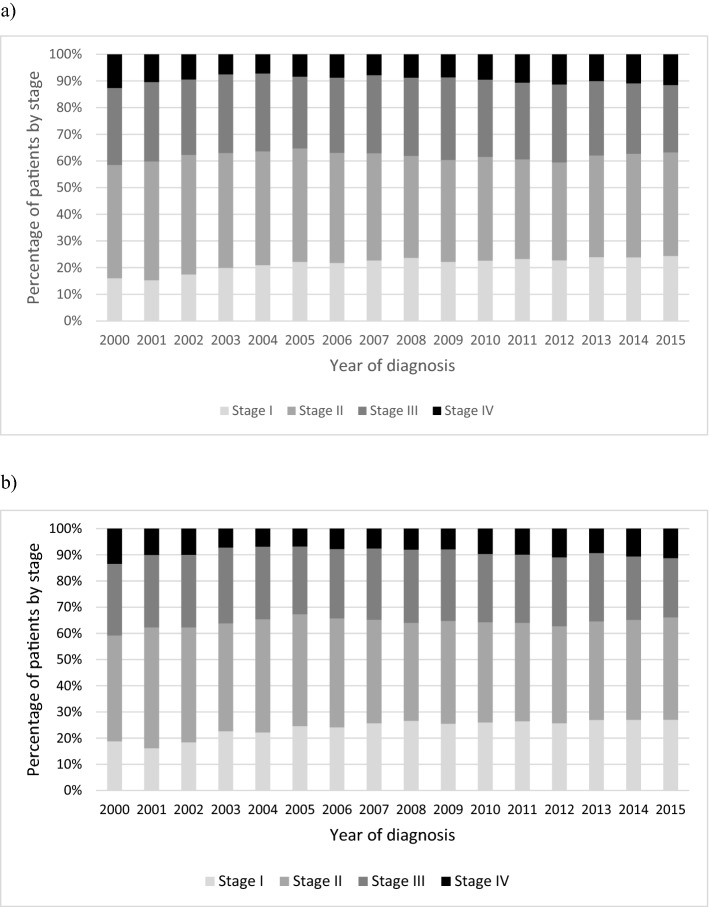
Percentage of clinical breast cancer stage at diagnosis by year of diagnosis. Cases included in the hospital-based cancer registries affiliated to the public health system in the state of Sao Paulo, Brazil. (**a**) All subjects; (**b**) screening-exposed population (50–69 years).

Figure 2 shows the timeline for clinical stage at diagnosis by year of diagnosis, when we considered all women (a) and the screening-exposed age group 50–69 (b). The proportion of subjects staged I slightly increased over time (Fig. 2a,b). There also seemed to be a slight decrease in the proportion of cases with tumors in more advanced stages (III–IV). These results may seem clearer when the screening-exposed population is considered alone.

Table 1 presents the descriptive analysis of data. Approximately 48% of cases were in the screening-exposed population, between 50 and 69 years at diagnosis. Considering only the cases with a known level of education (n = 56,237 cases), 67.2% of the cases had middle school or less. Carcinoma of no special type was the most frequent histology type (80.2%). Almost 53% of women received cancer care at hospitals located in their municipality of residence.

**Table 1:** Characteristics of female breast cancer diagnosed between 2000 and 2015 and included in the hospital-based cancer registries affiliated to the public health system in the state of São Paulo, Brazil.

Characteristics	Total		Stage I, II (n = 50,520)		Stage III, IV (n = 31,149)	
	N	%	N	%	N	%
**Age at breast cancer diagnosis (years)**						
< 40	8460	10.4	4526	53.5	3934	46.5
40–49	19,380	23.7	11,893	61.4	7487	38.6
50–59	21,596	26.4	13,680	63.3	7916	36.7
60–69	17,236	21.1	11,303	65.6	5933	34.4
70–79	10,595	13.0	6694	63.2	3901	36.8
≥ 80	4402	5.4	2424	55.1	1978	44.9
**Educational level**						
University	6529	8.0	4613	70.7	1916	29.3
High school	11,925	14.6	7558	63.4	4367	36.6
Middle school	13,306	16.3	8410	63.2	4896	36.8
Less than middle school	20,824	25.5	12,348	59.3	8476	40.7
Illiterate	3653	4.5	1863	51.0	1790	49.0
Data missing	25,432	31.1	15,728	61.8	9704	38.2
**Histologic type**						
Carcinoma of no special type	65,515	80.2	40,312	61.5	25,203	38.5
Lobular carcinoma	9321	11.4	5914	63.4	3407	36.6
Mucinous carcinoma	1096	1.3	855	78.0	241	22.0
Other	5737	7.0	3439	59.9	2298	40.1
**Travel for cancer care**						
No	43,050	52.7	27,203	63.2	15,847	36.8
Yes	38,619	47.3	23,317	60.4	15,302	39.6
**Year of diagnosis**						
2000–2003	15,441	18.9	9422	61.0	6019	39.0
2004–2007	17,703	21.7	11,246	63.5	6457	36.5
2008–2011	21,401	26.2	13,063	61.0	8338	39.0
2012–2015	27,124	33.2	16,789	61.9	10,335	38.1

Table 2 shows crude and multiple logistic regression. In the multiple regression, we observed that women with lower levels of education (Illiterate: OR 2.59; 95% CI 2.38–2.83; Less than middle school: OR 1.79; 95% CI 1.68–1.90) and those having to travel to receive BC care (OR 1.07; 95% CI 1.04–1.11) were more likely to be at an advanced stage at diagnosis. The results remained similar even when we considered multiple imputations in the multiple models.

**Table 2:** Crude and adjusted odds ratio (OR) for advanced female breast cancer by educational level and travel burden.

Characteristics	Complete records		Multiple imputations
	OR_crude_ [95% CI]	OR_adjusted_^a^ [95% CI]	OR_adjusted_^a^ [95% CI]
**Educational level**			
University	1.00 (ref.)	1.00 (ref.)	1.00 (ref.)
High school	1.39 [1.30; 1.48]	1.36 [1.28; 1.45]	1.30 [1.22; 1.38]
Middle school	1.40 [1.31; 1.49]	1.48 [1.38; 1.57]	1.43 [1.34; 1.52]
Less than middle school	1.65 [1.56; 1.75]	1.79 [1.68; 1.90]	1.75 [1.65; 1.87]
Illiterate	2.31 [2.13; 2.52]	2.59 [2.38; 2.83]	2.42 [2.22; 2.63]
**Travel for cancer care**			
No	1.00 (ref.)	1.00 (ref.)	1.00 (ref.)
Yes	1.13 [1.10; 1.16]	1.07 [1.04; 1.11]	1.07 [1.04; 1.10]

^a^Adjusted by all variables included in the table and also by age at diagnosis, histologic type, and year of diagnosis.

## Discussion

We report the characteristics of BC cases receiving cancer care at hospitals within the public health system in the state of São Paulo (Brazil), and we assessed the extent to which clinical stage at diagnosis was associated with some characteristics. Some significant findings have been made. First, our results show that a substantial proportion of women with BC receiving care at hospitals within the public health system are diagnosed at an advanced stage. Second, we found that women with lower levels of education and those having to travel to a different municipality to receive BC care have an increased chance of presenting with advanced BC stage at diagnosis. These findings have consequences in terms of cancer prevention policies.

The proportion of advanced BC stages at presentation observed in our study is worth comparing to previous publications. The prevalence of advanced stage at presentation (38.1%) is slightly lower than that observed in Brazil as a whole (40.2%)^7^. Nevertheless, the number is substantially different from what is observed in high-income countries (e.g., the US, 12.6% stage III–IV between 2004 and 2016; England, UK, 14.9% in 2015; Sweden, 17% between 1989 and 2013)^5,6,11^. Most high-income countries have systematic BC screening programs based on mammography, unlike low- and middle-income countries. Although the Brazilian Ministry of Health recommends biennial BC screening mammograms for women aged 50 to 69 years, since the early 2000s^12^, screening has only been offered on an opportunity basis, and the mammography coverage rate of the target population is heterogeneous among different geographic areas and subgroups^13,14^.

In their most recent global summit, the Breast Health Global Initiative continued to advise the promotion of early diagnosis in low- and middle-income countries before implementing mammography screening programs^8^. In this respect, the latest guidelines for early detection of BC in Brazil advise not only screening mammograms, but also recommend promoting early diagnosis, such as breast awareness policies, increased identification of suspicious signs and/or symptoms for prompt referral to specialized services, and one-stop diagnostic confirmation, as soon as possible^15^.

In our study, women with a lower level of education had a higher chance of advanced BC at diagnosis. Level of education has previously been shown to be associated with health literacy^16,17^ and with ability to access, understand, appraise and apply health information in decision-making regarding healthcare, disease prevention, and health promotion^18^. The association found here between lower levels of education and advanced stage at BC diagnosis is consistent with less participation of these patients in screening programs^13,14^ and longer intervals between the onset of BC signs/symptoms and first medical appointment^19^.

Another finding in the present study was that women receiving BC care outside their municipality of residence had an increased chance of advanced disease stage at diagnosis. One explanation could be barriers in access to BC diagnosis and treatment, as reported previously in Brazil^20–22^. More studies are required to better understand the impact of various factors in access to cancer care services in the state of São Paulo, such as the availability of these services within reach, geographic distance or travel time to them, other transportation difficulties, and financial constraints as reported in other countries^9,23^.

Since the advent of the SUS in 1988, a national cancer care network has been established, expanded, and improved in its integration^24–26^. However, there is still a deficit of accredited cancer services depending on the geographic area^25^. Using SUS data from 2014 to 2016, Saldanha and col^27^ reported that 51% of BC cases in Brazil were treated in a municipality different from that of residence, and the median time for those who need to travel to receive treatment was approximately 3 h. Other than that, in some regions of the country there are no standardized referral pathways and BC patients end up using a wide variety of pathways from the first appointment to first treatment^22^, which could contribute to delay in care.

It is essential to ensure that all women with BC have equal access to health care facilities. To improve cancer care, different BC patient navigation programs have been implemented across the world^28^, mainly aimed at increasing adherence to screening programs, and at earlier diagnosis and treatment. This strategy, still very incipient in Brazil, if pursued, should help reduce system-level barriers.

As far as we know, this is the first study to assess the effect of the need to travel for cancer care on BC stage at diagnosis in the state of São Paulo. This is also one of the largest studies performed in Brazil describing BC patient characteristics at diagnosis. However, this study presents limitations. Firstly, our study included only BC cases who received their first treatment at hospitals with oncology accreditation affiliated to the public health system in the state of São Paulo. Moreover, we do not know exactly what proportion of all cases diagnosed in the state of São Paulo between 2000 and 2015 were not included in our analysis. BC patients may also receive cancer care in general hospitals without oncology accreditation affiliated to the public health system and at private hospitals. In December 2020, approximately 22% of the Brazilian population (37% in the state of São Paulo) had private health insurance (available at www.ans.gov.br/anstabnet/cgi-bin/dh?dados/tabnet_br.def and https://www.ibge.gov.br/en/statistics/social/population/18448-estimates-of-resident-population-for-municipalities-and-federation-units.html?=&t=resultados), but many of these individuals tend to use the SUS for cancer treatment^29^. Secondly, unfortunately, the FOSP-HBCR does not include information regarding some tumour characteristics (e.g. histologic grade and molecular subtypes), patient details (e.g., ethnicity, menopausal status, nulliparity, family history of BC), or the date of the first contact with the health system. This information could help describe better the characteristics of subjects and contribute to better adjustment of the models. Finally, 31% of our sample presented missing information for level of education; however, we performed a sensitivity analysis using a multiple imputation model, and the results were very similar.

In conclusion, we identified a high proportion of advanced BC stages at diagnosis in our setting, with some groups (women with a lower level of education and those that had to travel for cancer care) having a higher chance of being diagnosed at these stages. Financing breast awareness programs and implementing pathways to accelerate BC diagnosis and treatment would be crucial to achieve early BC detection in most cases.

## Data Availability

The data that support the findings of this study are available from Fundação Oncocentro de São Paulo (FOSP). Restrictions apply to the availability of these data, which were used under license for this study. Data are available from the first author with the permission of FOSP.
